# New Documents about Lady Kahhal, First Female Ophthalmologist in Iran

**DOI:** 10.31661/gmj.v9i0.1898

**Published:** 2020-12-25

**Authors:** Seyyed Alireza Golshani, Babak Daneshfard

**Affiliations:** ^1^Department of History, Faculty of Literature and Humanities- Dr Ali Shariati, Ferdowsi University of Mashhad, Mashhad, Iran; ^2^Research Center for Traditional Medicine and History of Medicine, Shiraz University of Medical Sciences, Shiraz, Iran; ^3^Traditional Medicine Clinical Trial Research Center, Shahed University, Tehran, Iran

**Keywords:** Ophthalmology, Medical Journalism, History of Medicine, Iran, Lady Kahhal, Danesh Newspaper

## Dear Editor,

**Figure 1:**
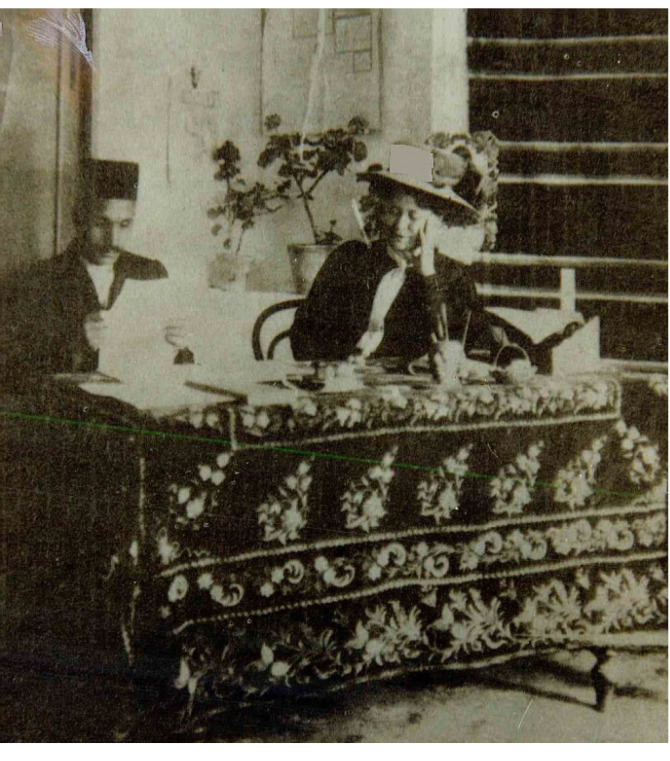
Lady Dr. Kahhal sitting in her newspaper office besides her son (Abbas Masoom), Tehran, 1910 (with permission from National Library and Archives of I.R. Iran)

**Figure 2:**
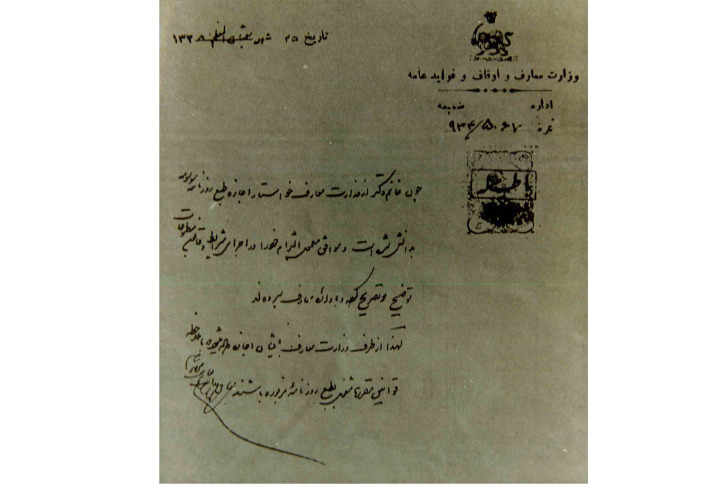
Letter by Morteza Gholi Khan Hedayat issuing the permission for publication of Danesh newspaper by Lady Dr. Kahhal, 1910 (with permission from National Library and Archives of I.R. Iran)

**Figure 3:**
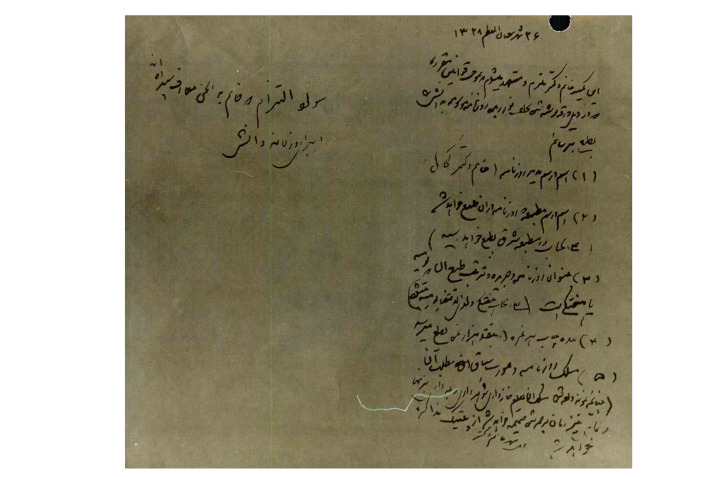
Legal obligation to publish Danesh newspaper, 1910 (with permission from National Library and Archives of I.R. Iran)

In a previously published paper [1], we had discussed about one of the prominent activities of women at the time of the constitutional revolution in Iran. Lady doctor Kahhal was the first female ophthalmologist in Iran who was visiting the patients as a physician and published the first specialized scientific newspaper, which was dedicated to women’s issues. “Danesh Newspaper,” first time published on September 14, 1910 in Tehran, included healthcare issues related to women and children, hygiene, and public health. In our recent investigations, we incidentally found some documents in National Library and Archives of I. R. Iran which provide new evidence for our claims in this regard and shed light on the whole story [2]. These documents have not ever been seen or published elsewhere. In an interesting picture, which seems to be the only remaining photograph of Dr. Kahhal (Figure-1), she is seen in the newspaper office while dressed like European people. The man sitting beside Lady Kahhal is her son, *Abbas Masoom*, helping her mother publish the Danesh newspaper. Another document (Figure-2) is a letter in which *Morteza Gholi Khan Hedayat* (1856–1911 AD), the first chairman of the Iranian parliament and minister of “Education and Endowments and Public Benefits” in Qajar dynasty, has issued the permission for publication of Danesh newspaper by Lady Dr. Kahhal on August 31, 1910. The last document (Figure-3) is the legal obligation to publish the Danesh newspaper dated September 1, 1910. These documents definitely confirm the publication of the Danesh newspaper by Lady Dr. Kahhal in 1910. Although the publication of this newspaper was ceased soon after its start, in 1911, the endeavors and activities of Iranian women for improvement and achieving their real position in the society just began.

## Conflict of Interest

 There is no conflict of interest.

## Acknowledgment

 The authors would like to thank the National Library and Archives of the Islamic Republic of Iran for the cooperation in providing the documents.
